# Xenograft Tumors Vascularized with Murine Blood Vessels May Overestimate the Effect of Anti-Tumor Drugs: A Pilot Study

**DOI:** 10.1371/journal.pone.0084236

**Published:** 2013-12-31

**Authors:** Zhihong Dong, Atsushi Imai, Sudha Krishnamurthy, Zhaocheng Zhang, Benjamin D. Zeitlin, Jacques E. Nör

**Affiliations:** 1 Angiogenesis Research Laboratory, Department of Restorative Sciences, University of Michigan School of Dentistry, Ann Arbor, Michigan, United States of America; 2 Department of Biomedical Engineering, University of Michigan College of Engineering, Ann Arbor, Michigan, United States of America; 3 Department of Otolaryngology, University of Michigan School of Medicine, Ann Arbor, Michigan, United States of America; 4 Comprehensive Cancer Center, University of Michigan, Ann Arbor, Michigan, United States of America; Bauer Research Foundation, United States of America

## Abstract

Recent evidence demonstrated that endothelial cells initiate signaling events that enhance tumor cell survival, proliferation, invasion, and tumor recurrence. Under this new paradigm for cellular crosstalk within the tumor microenvironment, the origin of endothelial cells and tumor cells may have a direct impact on the pathobiology of cancer. The purpose of this pilot study was to evaluate the effect of endothelial cell species (*i.e.* murine or human) on xenograft tumor growth and response to therapy. Tumor xenografts vascularized either with human or with murine microvascular endothelial cells were engineered, side-by-side, subcutaneously in the dorsum of immunodefficient mice. When tumors reached 200 mm^3^, mice were treated for 30 days with either 4 mg/kg cisplatin (i.p.) every 5 days or with 40 mg/kg sunitinib (p.o.) daily. Xenograft human tumors vascularized with human endothelial cells grow faster than xenograft tumors vascularized with mouse endothelial cells (P<0.05). Notably, human tumors vascularized with human endothelial cells exhibited nuclear translocation of p65 (indicative of high NF-kB activity), and were more resistant to treatment with cisplatin or sunitinib than the contralateral tumors vascularized with murine endothelial cells (P<0.05). Collectively, these studies suggest that the species of endothelial cells has a direct impact on xenograft tumor growth and response to treatment with the chemotherapeutic drug cisplatin or with the anti-angiogenic drug sunitinib.

## Introduction

It is generally agreed that animal xenograft tumors constitute an important model for studies on the pathobiology of tumors and for the testing of new anti-tumor drugs [1]–[3]. The xenograft tumor model was established in 1950s as a means to study human tumor cells by implanting them subcutaneously in immunodeficiency mice [4]–[6]. Since then, this model has been widely employed for mechanistic studies of tumor growth and progression, and for the screening of new anti-tumor agents. However, a retrospective study performed by the National Cancer Institute (NCI) revealed that the preclinical anti-tumor activity in human tumor xenografts animal models did not correlate with Phase II therapeutic activity in the clinical trials [7]. Considering the fact that human tumor xenografts are still widely used, it is imperative that we understand mechanisms involved in its shortcomings, and exploit new approaches to improve this experimental model.

It has been proposed that subcutaneous xenograft tumor models cannot reproduce the tumor microenvironment of human tumors [8]. Notably, the angiogenic vasculature is a critical component of the tumor microenvironment, and therefore much effort has been dedicated to the development of models of human angiogenesis [9]–[12]. We have worked with a model that involves the generation of xenograft tumors by the co-transplantation of human endothelial cells and human tumor cells seeded into biodegradable scaffolds [13]–[17]. This model allows for the establishment of human tumors vascularized with human vessels in immunodefficient mice. Using this model, we observed that endothelial cells initiate signaling events that directly influence tumor cell survival, proliferation, invasion and tumor recurrence [14], [18]–[22]. During the course of these experiments, we noticed that drugs that had a very significant anti-tumor effect in traditional xenograft models were not nearly as effective in the xenograft model with humanized vasculature. We thus hypothesized that maximal resistance to anti-tumor therapies is achieved when both endothelial and tumor cells are human. Here, we report data that demonstrate that xenograft tumors with humanized vasculature grow faster than xenografts vascularized with mouse vasculature and are more resistant to therapy with Cisplatin or Sunitinib, used here as models of a traditional chemotherapeutic and an anti-angiogenic drug.

## Materials and Methods

### Endothelial cells

Human dermal microvascular endothelial cells (HDMEC; Lonza, Walkersville, MD, USA) were cultured in endothelial cell growth medium (EGM2-MV; Lonza). Mouse dermal microvascular endothelial cells (MDMEC; Celprogen, San Pedro, CA, USA) were maintained in endothelial cell growth medium (Celprogen). HEK293T cells were co-transfected with the lentiviral packaging vectors psPAX2, pMD2.G, and the GFP expression vector pGIPZ (University of Michigan Vector Core, Ann Arbor, MI, USA) by the calcium phosphate method. HDMEC and MDMEC were infected overnight, selected with 1 µg/ml puromycin (Invitrogen, San Diego, CA, USA) for at least 1 week, and GFP expression was detected under fluorescence microscopy.

### Capillary sprouting assay

Endothelial cells (1.5×10^5^ cells/well) were cultured in 6-well plates containing a 1.5 ml layer of gelled type I collagen (Inamed, Santa Barbara, CA, USA), as described [17]. Cells were cultured in endothelial growth medium supplemented with 50 ng/ml VEGF (R & D Systems, Minneapolis, MN, USA). The number of capillary sprouts was counted daily in 6 random microscope fields (100×) from triplicate wells per condition. Here, and throughout this manuscript, studies were performed in triplicate samples/condition. At least 3 independent experiments were performed to verify reproducibility of the data.

### Flow cytometry

HDMEC and MDMEC cells were cultured in endothelial growth medium supplemented with 0–4 µM Cisplatin (Bedford laboratories, Bedford, OH, USA) or 0–4 µM Sunitinib (LC Laboratories, Woburn, MA, USA) for 48 hours. Cells were harvested and incubated in 50 µg/ml propidium iodide for 30 min for analysis of apoptosis and cell cycle.

### Sulforhodamine B (SRB) assay

HDMEC and MDMEC were plated in 96-well plates and cultured in growth supplemented with 0–100 µM cisplatin or sunitinib for 48 hours. Cells were fixed with a final concentration of 10% ice-cold trichloroacetic acid solution (TCA, Sigma) at 4°C for 1 hour, and stained with 50 µl of 0.4% sulforhodamine B solution for 30 min at room temperature. Absorbance was measured at 565 nm in microplate reader (GENious; TECAN, Austria).

### *In vivo* model of human tumor angiogenesis

Poly-L-lactic acid (PLLA; Boehringer Ingelheim, Ingelheim, Germany) biodegradable scaffolds measuring 6×6×1 mm were prepared, as described [13]. Briefly, 1.0×10^5^ tumor cells (UM-SCC-17B, HN12, or HeLa) and 9.0×10^5^ GFP-tagged endothelial cells (HDMEC or MDMEC) were seeded in each scaffold. Male 6- to 7- week-old CB-17 SCID mice (Charles River Laboratory, Wilmington, MA, USA) were anesthetized with ketamine/xylazine, and 2 scaffolds (one containing tumor cells and HDMEC and the other with tumor cells and MDMEC) were implanted bilaterally in the subcutaneous space of each mouse (8 mice in each cohort). When the average tumor size reached around 180 mm^3^, mice received 4 mg/kg cisplatin (i.p.) every 5 days or 40 mg/kg sunitinib (o.r.) daily. Tumor volume was evaluated daily by calipers by an investigator blinded for experimental groups, until the untreated controls reached an average volume of 1,000 mm^3^. Immediately after euthanasia, xenografts were retrieved and processed for histology. The identity of the tumor cell lines used here was confirmed by genotyping.

### Ethics statement

The care and treatment of mice were in accordance with University of Michigan's institutional guidelines, and under a protocol reviewed and approved by the University Committee of Use and Care of Animals (UCUCA). UCUCA is the Ethics committee from the University of Michigan responsible for the regulation of animal research. All surgical procedures were performed under anesthesia with Ketamine to minimize suffering.

### Immunohistochemistry

Tissue sections were deparaffinized with xylene, rehydrated using descending concentrations of ethanol, and then exposed to 0.1% triton X-100 (Fisher, Fair Lawn, NJ, USA) for 10 minutes at room temperature. Peroxidase activity was quenched with 1% H_2_O_2_ in methanol for 10 minutes at room temperature and then sections were treated with an antigen retrieval solution (Dako, Carpinteria, CA, USA) for 20 min at 90–95°C in a water bath. After two 10-minute washes with the Dako washing buffer (Dako), staining was done manually by incubation of the tissue sections in polyclonal rabbit anti-GFP (1∶500, Evrogen, Moscow, Russia) or rabbit anti-human Factor VIII antibody (1∶500, NeoMarkers, Fremont, CA, USA) overnight at 4°C followed by DAB staining (EnVision + Dual Link System-HRP, Dako) and counterstaining with hematoxylin. The number of microvessels in 10 random fields/implant was counted in 8 scaffolds per experimental condition. Alternatively, tissue sections were incubated with a mouse monoclonal antibody against p65, conjugated to FITC (1∶500, Santa Cruz Biotechnology, Santa Cruz, CA, USA) overnight at 4°C. In this case, cover slips were mounted using Vectashield with DAPI (Vector Laboratories, Burlingame, CA, USA). Images were taken with a Nikon E800 microscope with a camera (SPOT RT Slider; Spot Diagnostic) and processed with the SPOT RT 3.0 software.

### Statistical analysis

Data was analyzed by t-test or one-way ANOVA followed by the Student-Newman-Keuls test with SigmaStat 2.0 software (SPSS, Chicago, IL). Statistical significance was determined at p<0.05.

## Results

### Human and mouse endothelial cells generate similar vascular networks in xenograft tumors

**Figure 1:**
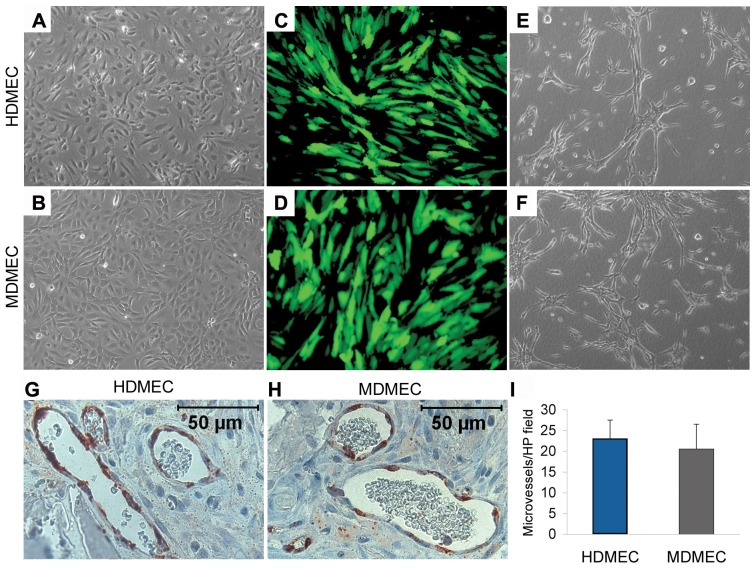
Human and mouse dermal microvascular endothelial cells have similar angiogenic potential *in vitro* and *in vivo*. (**A-D**) Representative photomicrographs of human dermal microvascular endothelial cells (HDMEC) and mouse dermal microvascular endothelial cells (MDMEC) stably transduced with GFP under light microscopy (**A** and **B**) and fluorescence microscopy (**C** and **D**). (**E** and **F**) Representative images of capillary sprouts formed by HDMEC (**E**) and MDMEC (**F**) on 3-D type I collagen matrices. (**G** and **H**) Photomicrographs of representative fields of GFP-immunostaining (red color) used to localize the blood vessels formed by HDMEC (**G**) and MDMEC (**G**) 14 days after implantation in immunodefficient mice. (**I**) The number of microvessels in implants populated with HDMEC or MDMEC. Microvessels were counted in 10 random microscopic fields/scaffold (200×) in 6 scaffolds per group. Results are representative of 3 independent experiments, n = 6.

Here, we used human dermal microvascular endothelial cells (HDMEC) and mouse dermal microvascular endothelial cells (MDMEC), which presented similar morphology under standard cell culture conditions (**Figure 1A and B**). HDMEC and MDMEC were stably transduced with lentivirus expressing green fluorescence protein (GFP) to allow for their identification after transplantation (**Figure 1C and D**). These cells showed similar capacity to form capillary-like sprouts when cultured in 3-D collagen matrices and treated with VEGF (**Figure 1E and F**). To evaluate their ability to form functional vessels *in vivo*, we seeded HDMEC or MDMEC stably transduced with GFP in biodegradable scaffolds and implanted them subcutaneously in immunodefficient mice. After 14 days, the scaffolds were removed and GFP immunohistochemistry was performed to confirm that the cells lining the blood vessels were indeed transplanted cells, and not mouse blood vessels that invaded the scaffold (**Figure 1G and H**). Microvessel density analysis showed that transplantation of human and mouse endothelial cells gave rise to similar numbers of microvessels (**Figure 1I**). Notably, blood cells were clearly visible in GFP-positive microvessels (**Figure 1G and H**), which confirms our previous observations that the engineered vasculature is functional and capable of connecting to host vessels through anastomosis [**13**].

### Tumor xenografts with humanized vasculature grow faster than xenografts with murine vasculature

**Figure 2:**
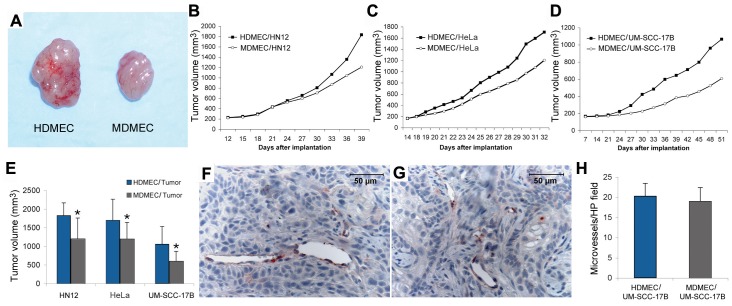
Tumor xenografts vascularized with human endothelial cells have similar microvessel density by grow faster than xenografts vascularized with mouse endothelial cells. (A) Representative photographs of HeLa tumors vascularized with either HDMEC or MDMEC. (B-D) Graphs depicting tumor growth of xenografts of HN12 (B), HeLa (C), UM-SCC-17B (D) vascularized with HDMEC or MDMEC. (E) Graph depicting tumor volume at the end of the experimental period. Asterisk depicts p<0.05. (F and G) Representative photomicrographs of tumors generated with UM-SCC-17B and HDMEC or MDMEC. Immunohistochemistry for GFP was performed to identify GFP-tagged endothelial cells. (H) Microvessel density of xenograft tumors generated with UM-SCC-17B and HDMEC or MDMEC. Results are representative of 3 independent experiments, n = 8.

To evaluate the impact of the species of endothelial cells on tumor growth, we co-implanted a diverse group of human tumor cells (UM-SSC-17B, HN12, or HeLa) with either HDMEC or MDMEC in immunodefficient mice. Surprisingly, we observed that in the three tumor cell models evaluated, the xenografts vascularized with human endothelial cells grew faster than the xenografts vascularized with mouse endothelial cells (**Figure 2A-E**). However, we did not observe a significant difference in tumor microvessel density when HDMEC and MDMEC tumors were compared (**Figure 2F-H**). Therefore, the difference observed in the rate of tumor growth is not simply due to having more blood vessels in the tumors with humanized vasculature. Collectively, these results imply that other factors besides simple access to blood and oxygen are playing a role in the growth of xenograft tumors.

### Human and mouse endothelial cells respond differently to a chemotherapeutic and an anti-angiogenesis drug *in vitro*

**Figure 3:**
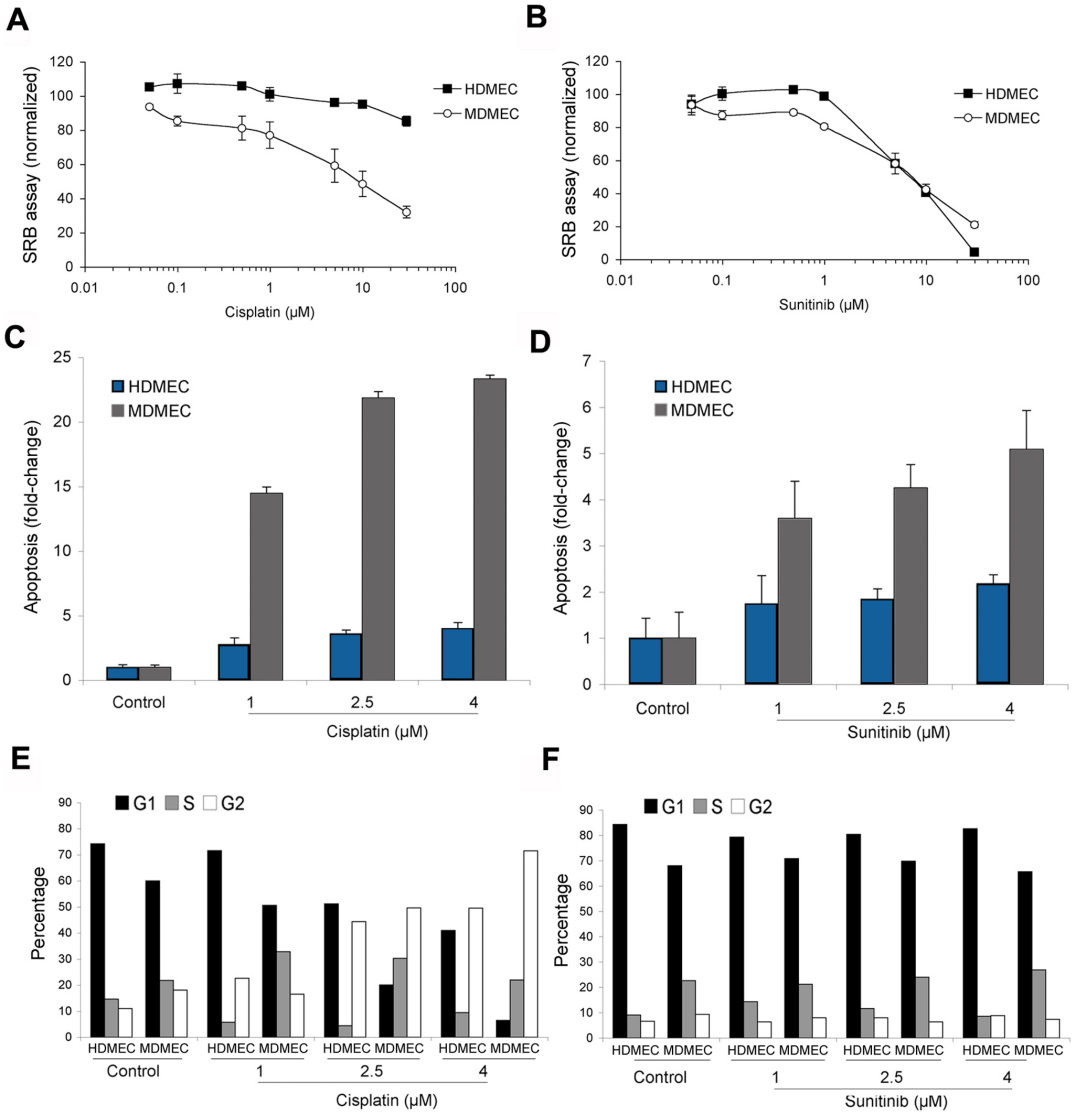
*In vitro* response of human and mouse endothelial cells to a chemotherapeutic and an anti-angiogenesis drug. (**A** and **B**) The 48-hour cytotoxicity of cisplatin (**A**) and sunitinib (**B**) was evaluated by the SRB assay in HDMEC and MDMEC. Results are normalized against vehicle control and initial plating density. (**C** and **D**) Fold-change difference in the percentage of apoptotic cells upon treatment with cisplatin (**C**) or sunitinib (**D**) for 48 hours. Apoptosis was determined as the percentage of cells in Sub-G_0_/G_1_ by propidium iodide staining followed by flow cytometry. (**E** and **F**) Effect of cisplatin (**E**) or sunitinib (**F**) on the cell cycle of HDMEC and MDMEC. Results are representative of 3 independent experiments.

To understand the impact of the species of endothelial cells on the response to anti-cancer drugs, we performed a series of studies using cisplatin as a model of chemotherapeutic drug and sunitinib as a model of targeted anti-angiogenic drug. Cytotoxicity assays showed that mouse endothelial cells are more sensitive to cisplatin than human endothelial cells (**Figure 3A**). In contrast, sunitinib was equally toxic to both mouse and human endothelial cells. As expected, cisplatin induced G2 cell cycle arrest and apoptosis in both human and murine endothelial cells (**Figure 3C and E**). However, the mouse endothelial cells were significantly more sensitive to induction of apoptosis by cisplatin than the mouse endothelial cells (**Figure 3C**). For sunitinib, we observed more apoptosis in the mouse endothelial cells than the human endothelial cells (**Figure 3D**). However, this effect was likely offset by the observation that the proportion of proliferating endothelial cells (*i.e.* cells in the “S” phase of cell cycle) was consistently higher than in the MDMEC group than in the HDMEC (**Figure 3F**). Collectively, these results demonstrated that the response of endothelial cells to anti-tumor drugs is complex and species-specific.

### Human tumor xenografts with humanized vasculature are more resistant to anti-cancer drugs than xenografts with murine vasculature

**Figure 4:**
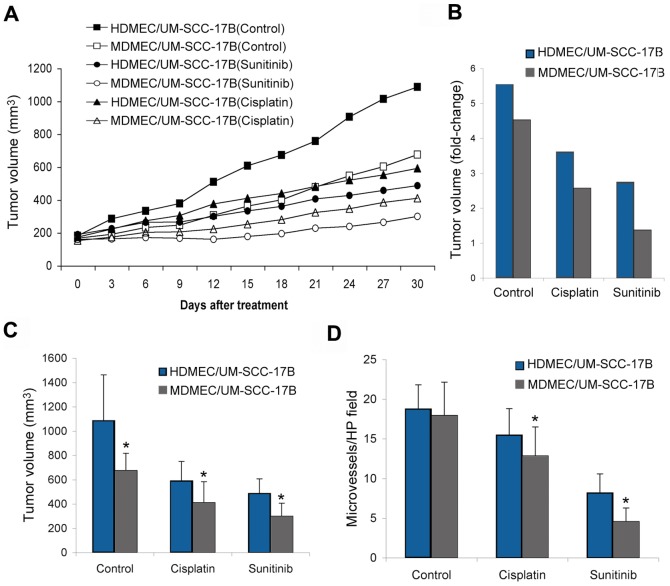
Human tumor xenografts vascularized with mouse endothelial cells is more responsive to a chemotherapeutic and an anti-angiogenesis drug. Xenograft tumors were engineered in immunodefficient mice by the co-transplantation of human tumor cells and human endothelial cells or human tumor cells and mouse endothelial cells seeded in biodegradable scaffolds measuring 6×6×1 mm. (A) Graph depicting the growth of human xenografts tumors (UM-SCC-17B cells) vascularized with HDMEC or MDMEC. As soon as we observed growth of the tumors beyond the size of the scaffold (*i.e.* when average tumor volume was 180 mm^3^), mice began to receive either 4 mg/kg cisplatin (i.p.) every 5 days, or 40 mg/kg sunitinib (o.r.) daily. (B) Fold change difference between the pre-treatment volume of the tumors and the volume of the same tumors after 30 days of treatment with cisplatin or sunitinib. (C) Tumor volume at the end of treatment with cisplatin or sunitinib. (D) Graph depicting the number of microvessels in tumor xenografts vascularized with HDMEC or MDMEC after treatment with cisplatin or sunitinib. Asterisk depicts P<0.05, as compared with controls. Results are representative of 3 independent experiments, n = 8.

Here, we co-transplanted human tumor cells with human or murine endothelial cells bilaterally in immunodeficient mice. When average tumor size reached 200 mm^3^, we began administration of either cisplatin or sunitinib. As expected, both cisplatin and sunitinib inhibited tumor growth (**Figure 4A-C**). Immunohistochemical analysis revealed that both cisplatin and sunitinib showed a more pronounced decrease in microvessel density when tumors were vascularized with mouse blood vessels (**Figure 4D**). These results correlate with the observation that the anti-cancer effect of both drugs was more pronounced in tumors vascularized with mouse vessels. Notably, if one compares tumor volume at the end of the experimental period (30 days) with pre-treatment volume, one concludes that sunitinib had a tumoristatic effect on tumors vascularized with mouse endothelial cells, *i.e.* the average growth of the of tumors was limited to approximately 1.2-fold increase in volume during the experimental period (**Figure 4B**). In contrast, sunitinib allowed for a 2.8-fold increase in the volume of tumors vascularized with human vessels in the same mice (**Figure 4B**). As expected, treated tumors are smaller than untreated for both, xenografts vascularized with human or mouse endothelial cells (**Figure 4C**). These experiments were repeated with a second cell line (HeLa) and similar trends were observed (data not shown).

Interestingly, the tumor growth inhibition in sunitinib-treated groups was more apparent than in the cisplatin-treated groups (**Figure 4A and B**). These results followed a different trend as compared to the *in vitro* cytotoxicity results (**Figure 3A and B**). This observation emphasizes the importance of combining mechanistic *in vitro* studies with the testing in animal models. Collectively, these data demonstrate that the response to anti-cancer therapy is significantly affected by the species of the endothelial cells that vascularize the xenograft tumors.

**Figure 5:**
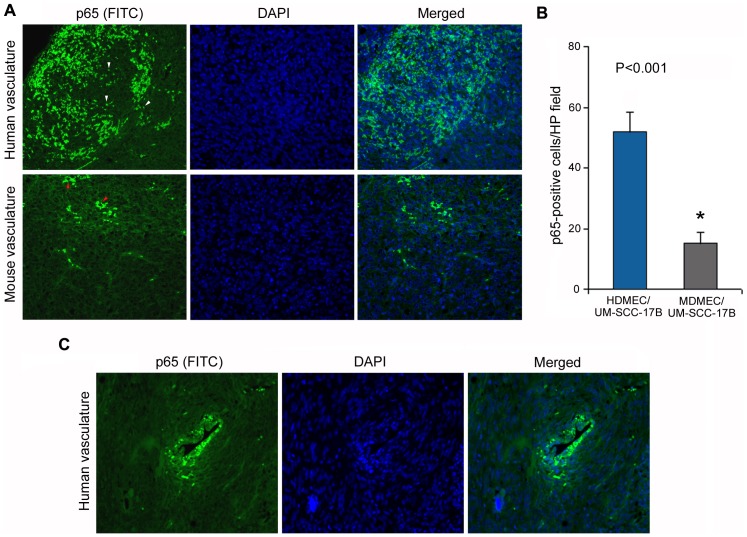
Nuclear expression of p65 in human xenografts vascularized with human endothelial cells. Xenograft tumors were engineered in immunodeficient mice by the co-transplantation of human tumor cells and human endothelial cells or human tumor cells and mouse endothelial cells. (A) Immunofluorescence imaging of p65-FITC (green) counterstained with DAPI (blue), and then merged. White arrowheads point to p65 expression in the nucleus in xenografts with humanized vasculature. Red arrowheads point to cytoplasmic p65 in xenografts with murine vasculature. (B) Graph depicting the number of p65-positive cells in tumor xenografts vascularized with HDMEC or MDMEC (n = 8) per high power field (400×). Asterisk depicts P<0.001. (C) Immunofluorescence imaging of p65-FITC (green) counterstained with DAPI (blue), and then merged of a human blood vessel in a xenograft tumor.

In attempt to explore possible mechanisms involved in the differential responses observed in xenografts with humanized vasculature, we interrogated the activity of the NF-kB pathway *in vivo*. It is well known that the NF-kB pathway is widely used by tumor cells as a regulator of genes that control cell proliferation and survival, and has a significant impact in resistance to therapy. Here, we observed higher levels of NF-kB activation (as measured by nuclear translocation of p65) in xenografts vascularized with human vessels (P<0.001), when compared to xenografts vascularized with mouse vessels (**Figure 5A and B**). Notably, this pathway is particularly active around blood vessels in xenografts with humanized vasculature (**Figure 5C**).

## Discussion

Several studies reported that the crosstalk between tumor cells and endothelial cells plays an important role in tumor angiogenesis and tumor growth. Wang and colleagues observed that the Notch ligand Jagged1 from head and neck squamous cell carcinoma (HNSCC) cells triggered Notch activation in neighboring endothelial cells and promoted capillary-like sprout formation [14]. Jagged1-expressing HNSCC cells significantly enhanced neovascularization and tumor growth *in vivo*. They concluded that the direct interplay between tumor cells and endothelial cells promotes tumor angiogenesis. We have reported that tumor cell-derived VEGF induces Bcl-2 expression in endothelial cells [23], and that the Bcl-2 expression levels in tumor endothelial cells correlate directly with the rate of tumor growth [18]–[19]. Notably, Bcl-2 acts as a signaling molecule by activating the NF-kB signaling pathway and inducing expression of CXCL1 and CXCL8 [24] that in turn enhance the invasive phenotype of neighboring tumor cells [21]. We also observed that endothelial cell-derived interleukin-6 (IL-6) and epidermal growth factor (EGF) induce the activity of the signal transducer and activator of transcription 3 (STAT3) and extracellular signal-regulated kinase (ERK) in head and neck cancer cells, resulting in enhanced tumor cell proliferation and protection against anoikis [22]. We speculate that the results reported here are mediated, at least in part, by the impact of species on the effectiveness of the molecular crosstalk between endothelial cells and tumor cells. An example of such differences might be the prominent role that CXCL8 has in the crosstalk between endothelial cells and tumor cells [22]–[24] and the well-known absence of this protein in murine cells [25]–[26].

It is well known that chemokines (*e.g.* CXCL8) are potent activators of NF-kB signaling [27]. NF-kB signaling plays a major role in cancer progression and on response to therapy [28]–[29]. Here, we observed that xenografts vascularized with humanized vessels express higher levels of nuclear p65 (indicative of active NF-kB signaling), when compared to xenografts vascularized with murine vessels. This difference in NF-kB activity is likely due to species-specific factors that affect the crosstalk between endothelial cells and tumor cells. Indeed, it is possible that the absence of CXCL8 in murine endothelial cells might contribute to the lower level of NF-kB activity in xenografts vascularized with murine endothelial cells. We speculate that the high NF-kB activity observed in xenografts with humanized vasculature might contribute to the faster tumor growth and to the resistance to therapy observed in these experimental tumors.

The approach used here involved the transplantation of a scaffold containing tumor cells and human endothelial cells in one side of the mouse, and transplantation of a scaffold containing the same tumor cells but mouse endothelial cells in the other side. This approach was designed to observe the potential impact of the species of endothelial cells on tumor growth and response to therapy with cisplatin and sunitinib, considering that the only variable was mouse *versus* human endothelial cells. We observed that human endothelial cells do not migrate outside the scaffolds and host endothelial cells (mouse vessels) do not migrate inside the scaffold within the timeframe of these experiments, as demonstrated by our studies with endothelial cells stably transduced with GFP. However, it is possible that soluble molecules secreted from the endothelial cells from one scaffold can enter the circulation and affect the behavior of the tumor generated in the other scaffold. We speculate that any effect of circulating molecules would minimize the potential impact of endothelial cell-species considering that it would affect both tumors. Therefore, the differences in tumor growth and response to therapy reported here are despite and beyond any potential long-distance effect mediated by endothelial cell-secreted molecules from different species transplanted in the same mouse.

A well-known quote from Dr. Judah Folkman is “if you have cancer and you are a mouse, we can take good care of you”. This statement reflects the observation that many drugs that are highly effective in curing experimental cancer in mice, have shown disappointing results in humans. In attempt to address this issue, the use of patient-derived xenograft (PDX) models in mechanistic and developmental therapeutics studies has increased over the last few years [30]. While PDX models demonstrate stability over a few passages in mice, a certain drift of stromal components has been observed with these models over time [30]. Also, we cannot exclude the possibility of a decrease in the fraction of human blood vessels using the scaffold model in long-term experiments. In recognition of such potential limitation, we give preference to short-term experiments (*e.g.* up to 2–3 months) with this model. Considering the importance of the tumor microenvironment to response to therapy, such drifts may hinder the translational potential of these preclinical models. It is clear that all preclinical tumor models have shortcomings, and that ideally one should use a combination of complementary approaches to enhance the predictive value of these methods for anti-cancer drug testing.

In conclusion, our studies demonstrated that xenograft human tumors vascularized with murine vessels are highly responsive to the anti-cancer drugs cisplatin and sunitinib. This might be due, at least in part, to the fact that traditional xenografts do not reproduce the effectiveness of the molecular crosstalk between endothelial cells and tumor cells, thereby making them more responsive to anti-cancer drugs. Collectively, this work supports the concept that a model that brings together tumor and endothelial cells of human origin offers a more rigorous approach for mechanistic studies of the tumor microenvironment and for translational studies involving the testing of anti-cancer drugs. It is imperative though that this pilot study is followed by larger studies, involving additional cell lines from several tumor types and multiple anti-tumor drugs to determine more definitely the impact of endothelial cell species on xenograft tumor growth and response to therapy. Nevertheless, the pilot data presented here suggest a potential explanation for the poor correlation frequently observed between the results of preclinical testing of anti-cancer drugs in traditional xenograft mouse models and the results of the clinical trials with these drugs in patients with cancer.
